# Design Validation of UWB MIMO Antenna with Enhanced Isolation and Novel Strips for Stop-Band Characteristics

**DOI:** 10.3390/e24060766

**Published:** 2022-05-30

**Authors:** Muhammad Kabir Khan, Quanyuan Feng

**Affiliations:** School of Information Science and Technology, Southwest Jiaotong University, Chengdu 611756, China; kabirnawab@my.swjtu.edu.cn

**Keywords:** WiMAX band, WLAN band, MIMO, UWB, X band

## Abstract

This article introduces a novel Ultra Wide Band (UWB) Multiple Input Multiple Output (MIMO) antenna with Triple-band notched characteristics. The overall dimensions of the antenna are 18 × 34 mm^2^. The designed antenna has two similar flower-shaped radiators with L-shape strips, common ground with two flag-shaped decoupling stubs and T-shape strips for notched band characteristics. Two flag-shaped stubs are used to achieve 22 dB improved isolation. The S_11_ of the designed antenna is less than −10 dB between 3.07 GHz and 12.40 GHz, having various stopped bands of WiMAX, WLAN and X bands. The presented antenna is examined and investigated in terms of S-parameters, Mutual Coupling, Gain, Envelope Correlation Coefficient (ECC), Efficiency and Diversity Gain (DG).

## 1. Introduction

In the recent past UWB antennas have gained extraordinary attention due to their channel capacity and high data rate. In UWB antennas there are some drawbacks of multipath fading [1,2]. To overcome the multipath fading problem, an effective solution like MIMO technology is presented. Combining UWB technology with MIMO antenna cannot only enhance the antenna’s properties such as channel capacity and transmission rate but also overcome the multipath fading problems [3]. Different shapes and techniques are presented in the literature for UWB communication and reduction of mutual coupling [4,5,6,7]. Some narrow bands already exist in the UWB range that cause interference, especially in WiMAX, WLAN, and X ranges. To avoid these interferences, a UWB antenna having a band–stop capability is needed. There are several methods to achieve stopband characteristics [8]. A Filtenna antenna with circuited stub is proposed for MIMO applications [9]. A CSRR is added to antenna components to gain dual notched band characteristics. The antenna has 3.10–10.65 GHz working bandwidth and secured isolation of 20 dB. The Filtenna antenna’s comprehensive dimensions are 68 × 35 mm^2^. A rectangular–shaped MIMO antenna having dual stopband characteristics is suggested [10]. The overall dimensions are 35 × 23 mm^2^. The antenna works from 3.10–10.90 GHz bandwidth except for notched bands and the improved isolation of the recommended antenna is 17 dB. The desired dual bands are stopped by notching slots in the antenna radiator. A MIMO antenna having band stopped properties is preferred [11]. The comprehensive diameter of the planned antenna is 26 × 35 mm^2^. The S11 of the fabricated antenna is <−10 dB from 2–10.60 GHz except for the desired notched bands and the antenna’s improved isolation is 22 dB. A UWB MIMO antenna is recommended [12]. The dimensions of the antenna are 18 × 36 mm^2^. The antenna’s operating bandwidth is 3–11 GHz, and the achieved isolation is 20 dB. The notched bands are obtained by extending a G-shaped stub from the ground plane. In [13], an inverted A-shaped monopole with DGS and orthogonal polarization is presented for enhanced isolation. The secured bandwidth is 3.10–10.60 GHz with a reduced mutual coupling of 17 dB. The antenna’s dimensions are 38.5 × 38.5 mm^2^ and a band-notched ability is obtained by adding different strips to the monopole antenna.

In [14], a circular antenna with U-shaped branches is proposed. The operating bandwidth is 2.82–11.2 GHz, and the secured isolation is 20 dB. To obtain the notched bands, an L-shaped slot in radiating components of the antenna is etched. A Cpw-fed Hammer-shaped MIMO antenna with Minkowski fractal DGS is presented in [15]. The submitted antenna’s dimensions are 26.75 × 41.5 mm^2^, and the improved isolation is 19 dB. The operating bandwidth is 3.1–11.5 GHz, and the notched bands are performed by etching C-shaped slots within radiating patches. A MIMO antenna with TVC–EBG is presented for triple notched characteristics in [16]. The covering bandwidth is 2–11 GHz with an improved isolation of 15 dB. The comprehensive size is 21 × 36 mm^2^.

In [17], a modified rectangular-shaped antenna with a stepped stub is presented for UWB application. The antenna operates from 3.08 to 10.98 GHz and has a port isolation of 20 dB. A notched band of 4.98–5.96 GHz is obtained by using the open-circuited stub. In [18], a Sierpinski antenna having a comprehensive size of 40 × 20 mm^2^ is proposed for UWB communication. The impedance bandwidth is 2.5–11 GHz with a notched WLAN band. The improved isolation is enhanced to 20 dB, and the notched band (5–6 GHz) is attained by using the U-shaped novel slot in the recommended antenna’s radiating components. A MIMO antenna having WLAN band-notched characteristic is introduced [19]. A novel vertical stub is initiated to maximize port isolation. The suggested antenna operates from 3.10 to 11 GHz. The achieved isolation is >18 dB, and the WLAN notched band is obtained by slotting a C-structured novel slot. A half-circle slot antenna is presented in [20]. The presented antenna’s size is 33 × 26 mm^2^ with an operating bandwidth of 3.10–10.60 GHz, and an open T-shaped slot is used to remove the WLAN interference. The secured isolation of the antenna is 15 dB. In [21], a half-circular and half-rectangular antenna having notched band properties are suggested for UWB application. The antenna’s dimensions are 20 × 36 mm^2^. The presented antenna works from 3.10 to 11.50 GHz with an achieved isolation of 21 dB. The authors have used a neutralization line for the enhancement of the isolation. In [22], an intended antenna having triple-notched properties is presented. The antenna’s size is 30.75 × 37.80 mm^2^, S11 < −10 dB and the desired bands are stopped by using fractal resonators. A Hilbert slot is etched to gain an isolation of 20 dB.

A 26 × 24.5 mm^2^ Vivaldi antenna having a dual-band rejection ability is proposed in [23]. To improve the insolation to 15 dB, a T-shaped slot is engraved between the antenna’s Vivaldi elements. SRR slits are used to achieve 5.10–5.90 GHz and 6.6–7.1 GHz rejection bands. The antenna works from 2.5 to 12 GHz. A vertically arranged monopole antenna with dual filtering ability is designed in [24]. The achieved frequency bandwidth is 3.10 to 10.60 GHz with 3.30–3.80 GHz and 5–6 GHz notched bands. The enhanced isolation of 20 dB is attained by including a parasitic element between the radiating elements. An 18 × 36 mm^2^ size MIMO antenna having a WiMAX stopped band is proposed in [25]. The 20 dB improved isolation is secured by inserting a rectangular-shaped parasitic structure. The recommended antenna operates in the 2.8–10.9 GHz bandwidth having a 3.4–3.9 GHz notched band. A monopole antenna having an F-shaped structure is proposed in [26]. The F-shaped antenna’s size is 40.5 × 40.5 mm^2^, and the impedance bandwidth is 3.10–10.6 GHz, having a 5.1–5.95 GHz notched band. SRR and DGS are used to obtain the enhanced isolation of 20 dB. A monopole antenna with a stepped ground is proposed in [27]. The antenna’s dimensions are 20 × 34 mm^2^, and the antenna works from 2.6 to 11.2 GHz frequency bandwidths. A parasitic element is used to achieve a high isolation of 20 dB. The WLAN band (5–6 GHz) is achieved by tuning the slot’s position and dimensions. A MIMO antenna with dimensions 35 × 52 mm^2^ is designed for UWB communication [28]. The antenna’s achieved bandwidth is 3.10–12 GHz, and 15 dB improved isolation is secured by the varying distance between two ground planes. A spatial MIMO antenna is experimentally investigated and proposed in [29]. Two L-shaped slits are utilized to achieve WLAN and C bands. The suggested antenna operating bandwidth is 2.9–10.8 GHz with 15 dB secured isolation, achieved by utilizing a T-structured stub. A miniature UWB MIMO antenna is designed [30]. The presented antenna’s radiating components consist of a circle with two ellipses. The size of the antenna is 40 × 22 mm^2^, and the working bandwidth is 3.18–11.26 with notched bands. The T-structured stub between the radiating elements is used to obtain 15 dB isolation. The detailed comparability of the presented design is conducted in Table 1 in terms of size, operating frequency, notched bands, mutual coupling, gain and ECC.

In the recommended research article, two ports MIMO antenna with flower-shaped radiating elements are designed. The UWB application is obtained by two flower-shaped radiating elements with common ground. Two flag-shaped stubs are applied to the ground to improve isolation. The antenna has a greater bandwidth of 3.07–12.40 GHz and has improved isolation of 22 dB. An extra novel T-shaped strip is joined to the antenna ground to remove the interference of the WiMAX (3.28–4.20 GHz) and X bands (7.35–7.76 GHz). Another L-shaped strip is connected to the flower-shaped radiating elements to remove the interference of WLAN (5.18–5.92 GHz).

## 2. Design and Investigation of the Designed Antenna

**Figure 1:**
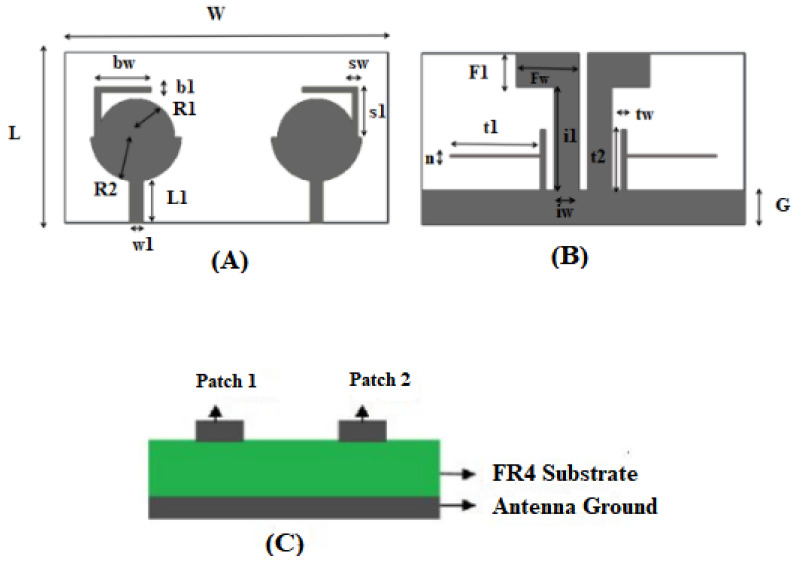
(**A**) Front, (**B**) back and (**C**) Side views of the suggested antenna.

To elaborate the MIMO antenna further, it is printed on an easily available 1.6 mm-thick FR4 substrate with relative permittivity = 4.4 and loss tangent = 0.02. The compact antenna size is 18 × 34 × 1.6 mm^3^. The flower-shaped radiating element is composed of two half circles to adjust the lower frequencies. Two flag-shaped stubs extruded from the antenna ground plane are used to minimize mutual coupling, as given in Figure 1. A T-shaped strip is added to the ground to secure the WiMAX and X stopped bands, and an L-shaped strip is extruded from the flower-shaped radiating element to stop the inference of the WLAN. The common ground between the radiating elements is practiced for size minimization.

**Figure 2:**
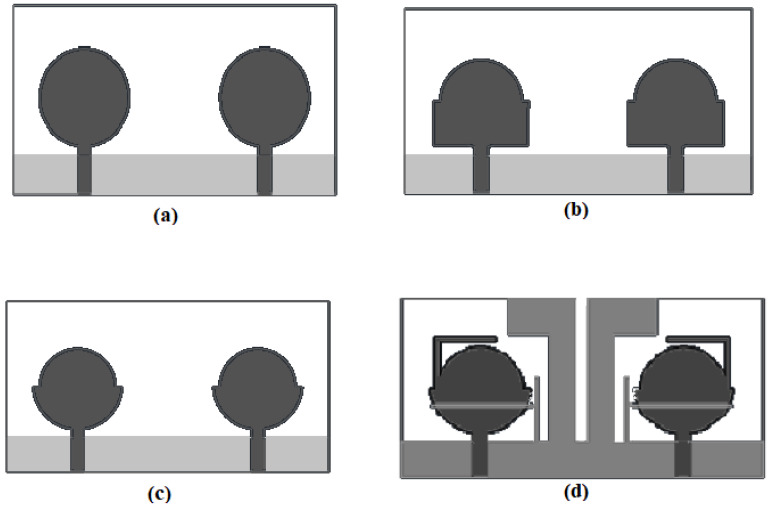
Designing of the Proposed MIMO Antenna: (**a**) circular-shaped antenna, (**b**) half circular-rectangular shaped antenna, (**c**) flower-shaped antenna and (**d**) flower-shaped antenna with band-notched strips and Flag-shaped stubs.

**Figure 3:**
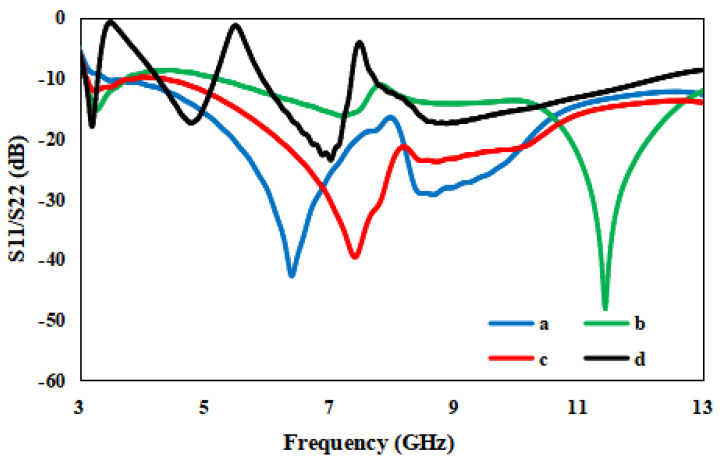
S-parameters for (a) circular-shaped antenna, (b) half-circular–rectangular shaped antenna, (c) flower-shaped antenna and (d) flower-shaped antenna with band-notched strips and flag-shaped stubs.

The radiating components of the suggested antenna are composed of two half circles of radii R1 and R2 and a novel L-shaped strip. The radiating element is joined by a 50 Ω micro strip line. The ground plane is shared by both radiating elements for size minimization with a dimension of W × G, adding two flag-shaped stubs to the conventional ground for securing enhanced isolation between two ports. An extra novel T-shaped strip is joined to the conventional ground to remove the interference of the 3.28–4.20 GHz WiMAX band and the X band (7.35–7.76 GHz). The novel L-shaped strip is added to the flower-shaped radiating element to stop the band of WLAN (5.18–5.92 GHz). To examine the antenna design further, four radiating models are designed and optimized in CST Microwave Studio, as shown in Figure 2. The final model is achieved after various steps: In the first step, a simple circular radiator is modeled that works from 3.6 GHz to 12 GHz, which is an unacceptable range for UWB as confirmed from Figure 3. The design is further modified to a half-circular–rectangular shaped antenna and a flower-shape antenna in step 2 and step 3, respectively, to achieve the entire UWB band.

The half-circular–rectangular-shaped antenna performs from 3.2 to 12 GHz. The rectangular-half-circular-shaped antenna’s lower edges are chambered, and the antenna is converted to a flower-shaped antenna to secure the entire UWB range. The results for the flower-shaped antenna are examined in Figure 3, which illustrates that the flower-shaped antenna operates from 3.07 to 12 GHz. In the next step, the effect of the flag-shaped stubs and the T- and L-shaped strips are examined. In the frequency range of 3.07–12.40 GHz, the S11 of the planned antenna is below −10 dB, and it also excludes the WiMAX band (3.28–4.20 GHz), WLAN band (5.18–5.92 GHz) and the X band (7.35–7.76 GHz).

**Figure 4:**
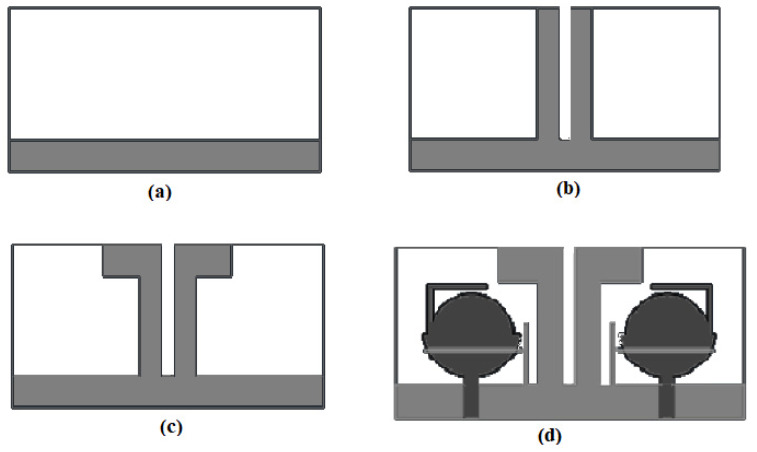
Transformation of decoupling geometry of the presented Antenna: (**a**) conventional ground, (**b**) I−shaped stubs, (**c**) flag−shaped stubs and (**d**) flag−shaped stubs with the band-notched strips.

The model evaluation of the decoupling stub is depicted in Figure 4. First of all, a conventional antenna ground is created and examined. The conventional ground is transformed further, two I−shaped stubs are added and the results are examined. After examining the results, further modification is needed, and it is modified to a flag−shaped structure to obtain a desirable mutual coupling.

**Figure 5:**
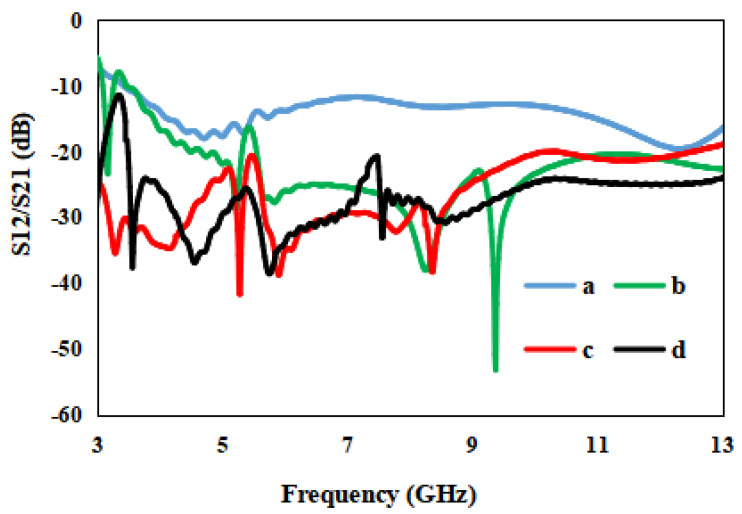
S12/S21 of the presented antenna for various stub structures: (a) without stubs, (b) I-shaped stubs, (c) flag–shaped stubs and (d) flag–shaped stubs with band-notched strips.

The conventional ground is investigated in terms of the transmission coefficient (S_12_/S_21_). The antenna with the conventional ground has a very low isolation of 12 dB, justified in Figure 5. To overcome the problem of low isolation, two I-shaped stubs of the same size are attached to the ground, and the mutual coupling is analyzed. The isolation is not sufficient, so an I-shaped stub is further modified to a flag-shaped stub to enhance the isolation to 22 dB, justified in Figure 5. Finally, a T-shaped strip is added to the conventional ground and an L-shaped strip is added to the radiator, and the effects of these strips and stubs on the designed antennas are investigated. The effect of the novel strips on the mutual coupling is nominal, and it is nearly the same, only a little shift at lower frequencies.

**Figure 6:**
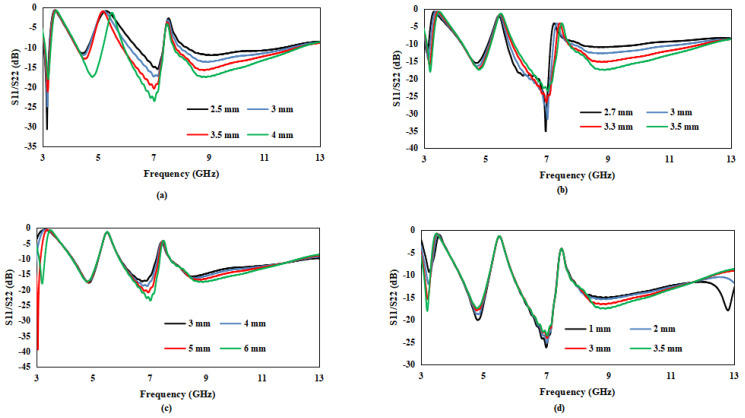
S11/S22 of the presented antenna: (**a**) Results of the presented antenna having different values of the circle having radius R1, (**b**) results of the presented antenna having different ground sizes, **(c**) results of the presented antenna having different vertical (I-shaped) stub sizes and (**d**) results of the presented antenna having different horizontal (FW) stub sizes.

The presented antenna’s comprehensive parametric analysis is carried out in this section. The radius of circle R1 with different values is examined. The effect of the circle radius R1 size on the proposed antenna’s S11 is clear from Figure 6a. The result with a radius of 4 mm is better than the other sizes for the complete range of UWB, as justified by Figure 6a. In Figure 6b, the effect of ground size on the reflection coefficient is investigated, and it is justified in Figure 6b that when the ground size is 3.5 mm, it gives a better result in the complete UWB range. The effect of vertical (I-shaped) stubs on antenna performances is shown in Figure 6c. Figure 6c confirms that when the vertical stub size is optimized to 6 mm, its results improve in the whole UWB range. To obtain better results, the effect of horizontal (Fw) stub on the antenna’s S-parameters is investigated and examined in Figure 6d. The results of the horizontal stub with a size of 3.5 mm are better in the complete UWB range.

The final parameters dimensions are measured in mm and mentioned in Table 2.

The T-shaped strip is joined to the conventional ground to remove the interference of the WiMAX band (3.28–4.20 GHz) and the X band (7.35–7.76 GHz). The novel L-shaped strip is added to the flower-shaped radiating element to stop the WLAN band (5.18–5.92 GHz). The dimensions of the strips are optimized so that the required UWB performance and the performance of the notched frequencies are not affected. To gain the desired frequency notches, each strip length is calculated from the given equation [31]:

(1) L=c4fn(εr+1)2

where $f_{n}$ is the notched frequency, *c* is the speed of light and *ε*_r_ is the relative permittivity.

**Figure 7:**
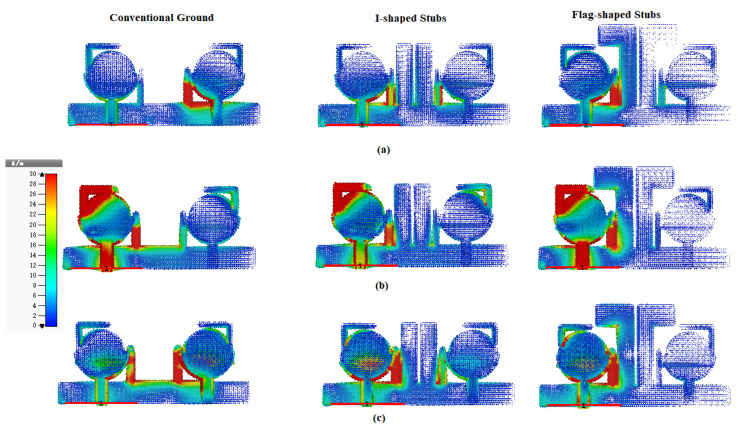
Current distribution with the conventional ground, I-shaped stubs and Flag-shaped stubs at (**a**) 3.50 GHz, (**b**) 5.50 GHz and (**c**) 7.50 GHz frequencies.

The current distribution is analyzed and examined at 3.5, 5.5 and 7.5 GHz to investigate the behavior of the notched strips and decoupling stubs by exciting port 1. The planned antenna’s current distribution is tested with a conventional ground plane in the first stage. A dense current distribution is observed at port 2 at all three frequencies (3.5, 5.5 and 7.5 GHz). In the next step, to minimize the dense current distribution at port 2, two I-shaped vertical stubs are added to the conventional ground plane as clear from Figure 7. After adding the I-shaped stub, the maximum current is observed at port 1, radiating element and I-shaped stub; the current distributed at port 2 is reduced, resulting in enhanced isolation than the conventional ground. To minimize the current distribution further, the I-shaped stub is modified to a flag-shaped stub. By adding the flag-shaped stub, the maximum current is observed at port 1, the radiating element and the flag-shaped stub, as justified in Figure 7. The current distribution at port 2 is minimized further as compared to the I-shaped stub, thus resulting in enhanced port isolation.

**Figure 8:**
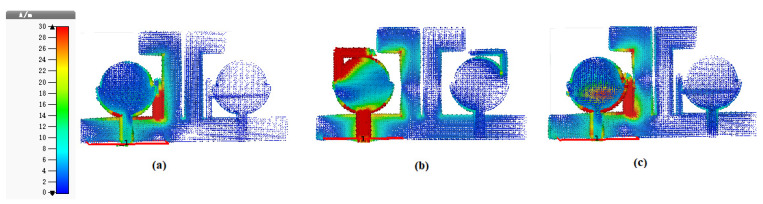
(**a**) The Fabricated antenna’s Current distribution having T-shaped strip at 3.50 GHz, (**b**) L-shaped strip at 5.5 GHz and (**c**) T-shaped strip at 7.5 GHz.

The printed antenna’s current distribution is analyzed further, and the effect of T- and L-shaped strips in rejecting bands are carried out separately. At stopped band 3.50 GHz, the strongest current is located at a T-shaped strip, resulting in a deep WiMAX band as is clear from Figure 8a. As is clear from Figure 8b, the maximum current at 5.5 GHz is directed to the L-shaped strip, resulting in a deep WLAN notched band. In Figure 8c, the current distribution is given at a frequency of 7.5 GHz, and the dense current is observed at the T-shaped strip, resulting in a stopped X band, justified in Figure 8c.

## 3. Results and discussion

**Figure 9:**
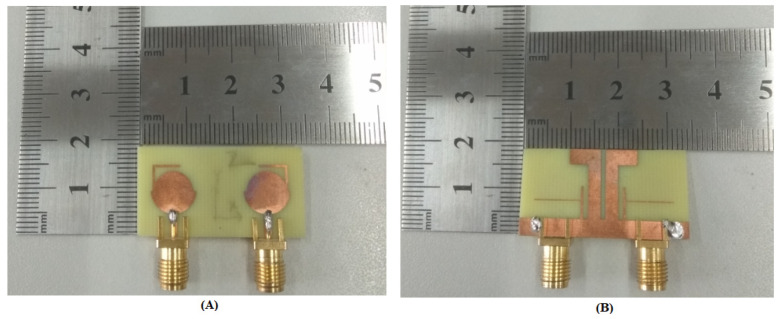
The designed MIMO Antenna’s printed prototype; (**A**) top and (**B**) bottom view.

The recommended flower-shaped antenna’s front and backside after fabrication are given in Figure 9. The results such as the reflection coefficient, transmission coefficient, radiation pattern, radiation efficiency, total efficiency, peak gain and diversity performance are discussed in detail.

### 3.1. Reflection Coefficient and VSWR

**Figure 10:**
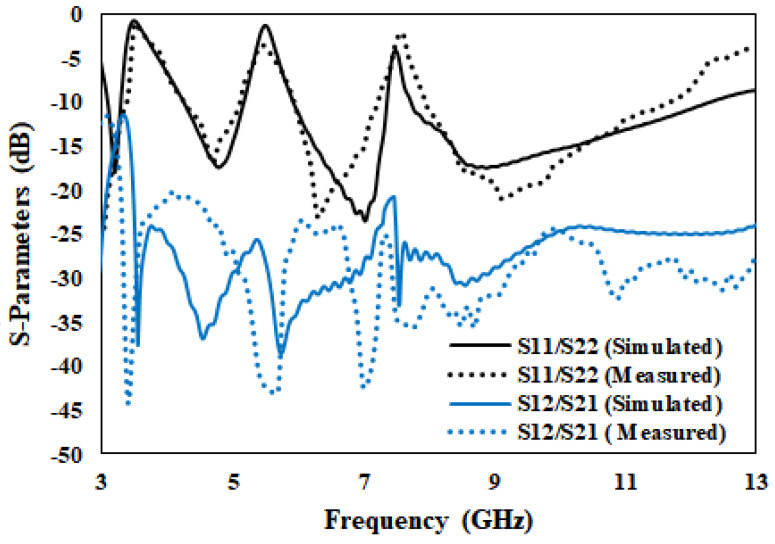
Simulated and Measured S-Parameters of the proposed antenna.

The flower-shaped antenna is validated by fabrication of a prototype and various results are measured such as the S-parameters, radiation pattern, peak gain, total efficiency and radiation efficiency. The measured and simulated S11< –10 dB from 3.07 to 12.40 GHz, except for WiMAX (3.28–4.20 GHz), WLAN (5.18–5.92 GHz) and (7.35–7.76 GHz) X notched bands. As confirmed from Figure 10, both results are nearly the same in the entire UWB range. The T-shaped strip is joined to the conventional ground to remove the interference of the WiMAX (3.28–4.20 GHz) and X bands (7.35–7.76 GHz). Another L-shaped strip is connected to the flower-shaped radiating elements to remove the interference of WLAN (5.18–5.92 GHz). The proposed antenna isolation is enhanced to 22 dB by connecting a flag-shaped stub to the ground.

**Figure 11:**
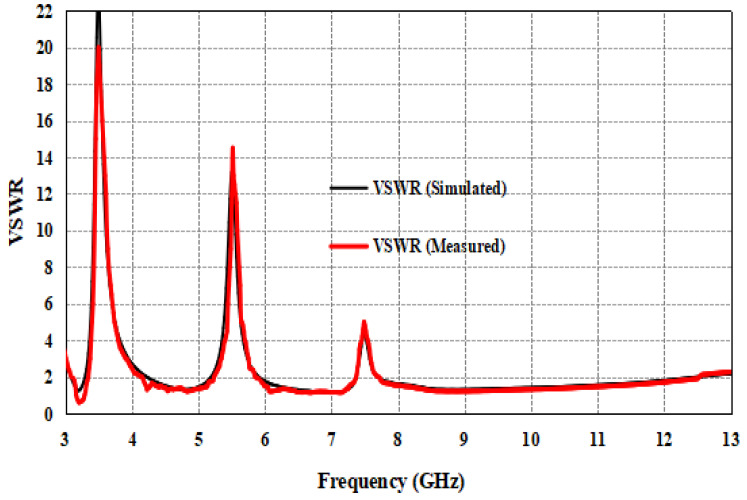
Simulated and measured VSWR of the proposed UWB antenna.

The simulated and measured VSWR of the proposed antenna is <2 in the frequency range of 3.07 to 12.40 GHz, except the notched bands. Both simulated and measured results are nearly the same. It can be observed from Figure 11 that the VSWR is 20 at 3.5 GHz, 14 at 5.5 GHz and 5 at 7.5 GHz notched bands. It is clear from Figure 10 and Figure 11 that the proposed antenna gives a better impedance bandwidth (S11 < −10 dB and VSWR < 2) in the entire UWB spectrum.

### 3.2. Radiation Patterns, Radiation Efficiency, Total Efficiency and Peak Gain

**Figure 12:**
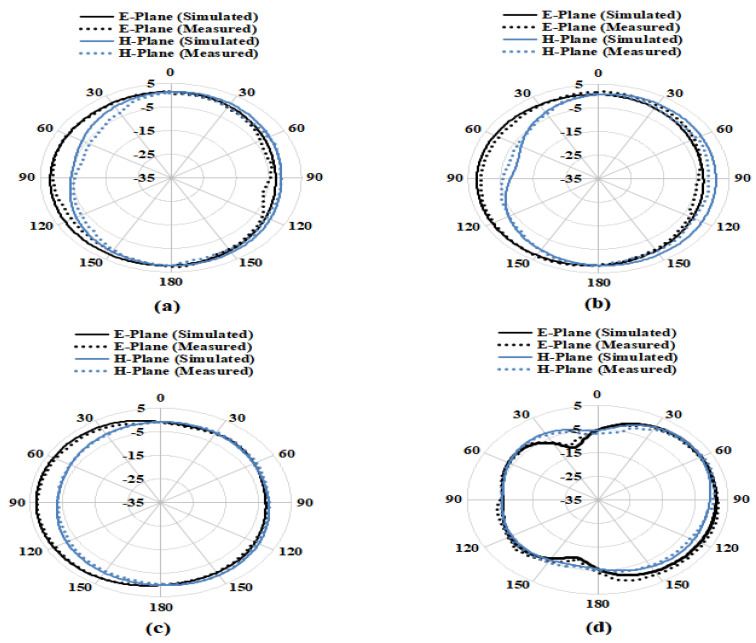
Simulated and Measured Radiation Patterns at (**a**) 4.5 GHz, (**b**) 6.5 GHz, (**c**) 8 GHz and (**d**) 12 GHz.

The designed antenna’s radiation efficiency, directivity, gain and radiation pattern are investigated in Satimo Star Lab (650 MHz to 18.0 GHz) anechoic chamber. This system allows for the measurement of the antenna’s electric fields in the near field region to compute the antenna under test (AUT) far field values. The AUT is placed in the center of a circular “arch” that contains 16 measuring probes on the test board. These measuring probes are placed at an equal distance surrounding the circular surface. The AUT horizontally rotates at 360 degrees, and this horizontal rotation and the 16 measuring probes together perform a complete 3D scan of the AUT for collecting data for 3D radiation patterns. By using SatEnv software, the near field data are transformed into far field data. The antenna gain, efficiency, directivity and radiation pattern are also estimated using the SatEnv software using the far field data [32,33]. A good resemblance is found between the simulated and measured radiation patterns of the presented antenna. The proposed antenna radiation performances are studied at 4.5 GHz, 6.5 GHz, 8 GHz and 12 GHz, as shown in Figure 12. The radiation patterns (both simulated and measured) at these frequencies are stable and nearly omnidirectional.

**Figure 13:**
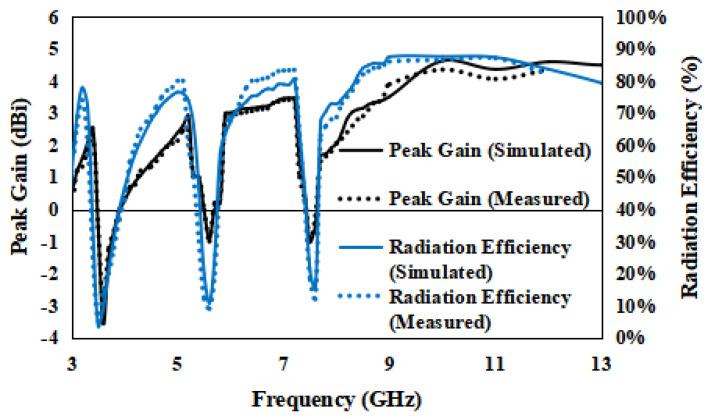
Radiation efficiency and peak gain of the planned antenna.

The antenna’s peak gain and radiation efficiency are given in Figure 13. The antenna peak gain is from 1.6 to 4.6 dBi, excluding the notched bands.

An antenna’s realized gain (G_R_) is the antenna gain reduced by power lost to reflections at the antenna feeding. The antenna gain is defined as the product of antenna efficiency (E_A)_ and directivity (D):

G_R_ = (1 − |S11|^2^) E_A_ D (2)

The antenna’s radiation efficiency is 78–87%, as is clear from Figure 13.

**Figure 14:**
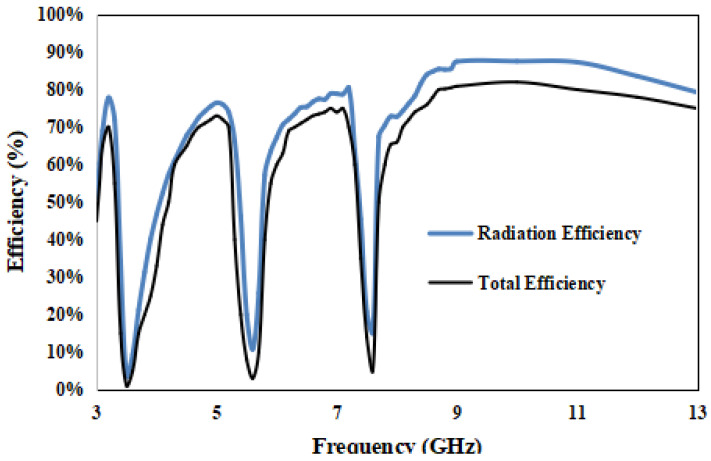
Radiation and total efficiency of the proposed antenna.

The radiation and total efficiency of the proposed antenna are given in Figure 14. The radiation efficiency of the proposed antenna is from 78 to 87%. The total efficiency takes feed network losses, ohmic losses and impedance mismatch into account. It can be observed from Figure 14 that the total efficiency of the proposed antenna is 70–80% in the entire operating bandwidth, except for the notched bands.

### 3.3. Diversity Performances of the Presented Antenna

To study the proposed antenna accuracy further, the diversity performances of the suggested antenna are evaluated. Ideally, the ECC value is zero, and a practical limit of 0.5 is acceptable. The ECC and the designed antenna’s DG are calculated using the following equations [34]:

(3) $$\mathrm{ECC}=\frac{|S_{11}^{*}S_{12}+S_{21}^{*}S_{22}{|}^{2}}{\left(1-|S_{11}{|}^{2}-|S_{21}{|}^{2})(1-|S_{22}{|}^{2}-|S_{12}{|}^{2}\right)}$$

(4) $$\mathrm{DG}=10\sqrt{1-{\left(ECC\right)}^{2}}$$

**Figure 15:**
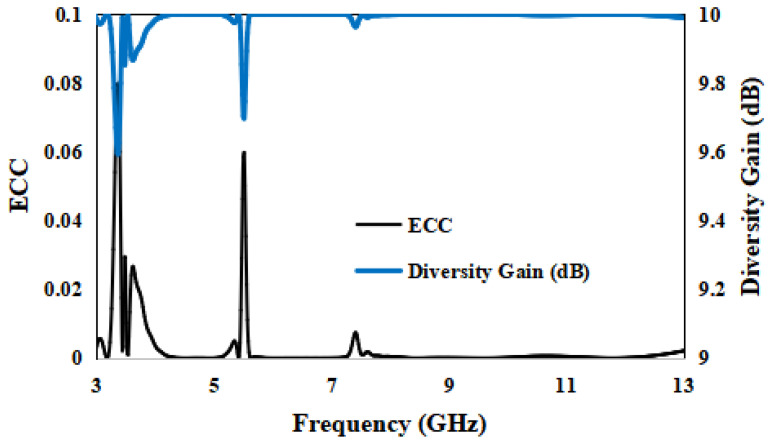
ECC and Diversity Gain of the designed antenna.

Generally, the acceptable limit of ECC is 0.5 and the ECC of the presented antenna is <0.001, except for the notched bands, as expected. The ECC at the notched bands is 0.08 at 3.5 GHz, 0.06 at 5.50 GHz and 0.01 at 7.50 GHz, as is clear from Figure 15. The Diversity Gain is >9.99 dB for the resonance frequencies, except the stopbands 3.5 GHz, 5.5 GHz and 7.5 GHz. The DG is 9.6 dB at 3.50 GHz, 9.7 dB at 5.50 GHz and 9.96 dB at 7.50 GHz, which is very low, as expected for the notched bands.

## 4. Conclusions

In the recommended research article, two ports flower-shaped MIMO antenna are designed to achieve UWB application with triple-notched band characteristics. The proposed antenna can avoid the interference from WiMAX (3.28–4.20 GHz), WLAN (5.18–5.92) GHz and X bands (7.35–7.76 GHz). The notched-band characteristics are obtained by introducing two novel T- and L-shaped strips. Two flag-shaped stubs are used to obtain the improved isolation of 22 dB. The proposed antenna achieves a peak gain from 1.6 to 4.6 dBi and a radiation efficiency of 78 to 87%. Antenna performances such as ECC, diversity gain, radiation patterns, gain, radiation efficiency and current distribution show that all the performance parameters are appropriate and the suggested antenna is a potential candidate for the UWB communication systems.

## Figures and Tables

**Table 1:** Comparison of the recommended antenna with previous work.

Ref.	Size (mm^2^)	Bandwidth (GHz)	Band Notches (GHz)	Isolation (dB)	Gain (dBi)	ECC
[11]	26 × 35	2–10.6	3.3–3.7, 5.17–5.25	<22	3.5	<0.2
[12]	18 × 36	3–11	3.25–3.75, 5–5.9	<20	−4–4	0.05
[13]	38.5 × 38.5	3.1–10.6	3.9–4.2, 5.1–5.85,	<17	0–6	0.035
[14]	25 × 39	2.82–11.2	5.08–5.83, 7.02–7.98	<20	2–5	0.01
[15]	26.75 × 41.5	3.1–11.5	3.3–3.7, 3.7–4.2, 5.15–5.85	<19	-	0.01
[16]	21 × 36	2–11	3.3–3.7, 5–6, 7.9–8.4	<15	0–7	0.015
[17]	30 × 30	3.08–10.98	4.98–5.96	<20	5	<0.13
[18]	40 × 20	2.5–11	5–6	<20	3	<0.1
[19]	29 × 40	3.1–11	5.725–5.825	<18	1.6–6.2	<0.02
[20]	33 × 26	3.1–10.6	4.5–5.5	<15	1–6	0.03
[21]	20 × 36	3.1–11.5	5.45–5.85, 7.15–7.95	<21	1.8–3	<0.19
[22]	30.75 × 37.80	2.7–11.22	3.7–4.2, 5.15–5.825, 7.9–8.4	<20	0.07–3.4	0.035
[23]	26 × 24.5	2.5–12	5.1–5.9, 6.6–7.1	<15	……	0.02
[29]	26 × 28	2.9–10.8	5.15–5.86, 6.7–7.1	<15	1.6–4	0.08
[30]	40 × 22	3.18–11.26	3.3–3.99, 4.97–5.93	<15	0–5.4	0.002
Proposed work	18 × 34	3.07–12.40	3.28–4.20, 5.18–5.92, 7.35–7.76	<22	1.6–4.6	<0.001

**Table 2:** Dimension of the antenna in mm.

Parameters	Size (mm)	Parameters	Size (mm)
L	18	G	3.5
W	34	R1	4
R2	4.7	L1	4.74
w1	1.3	s1	5
sw	0.5	b1	0.5
bw	4.8	i1	11
iw	2.5	F1	3.5
Fw	6.5	t2	6.5
tw	0.5	t1	10

## Data Availability

All the measured and simulated data are plotted for each parameter as a comparison.
